# Computational synchronization of microarray data with application to *Plasmodium falciparum*

**DOI:** 10.1186/1477-5956-10-S1-S10

**Published:** 2012-06-21

**Authors:** Wei Zhao, Justin Dauwels, Jacquin C Niles, Jianshu Cao

**Affiliations:** 1 Singapore-MIT Alliance for Research and Technology, Centre for Life Sciences, 28 Medical Drive, Singapore 117456; 2 School of Electrical and Electronic Engineering, Nanyang Technological University, 50 Nanyang Avenue, Singapore 639798; 3 Department of Biological Engineering, Massachusetts Institute of Technology, 77 Massachusetts Avenue, Room 56-341, Cambridge MA 02139, USA; 4 Department of Chemistry, Massachusetts Institute of Technology, 77 Massachusetts Avenue, Room 6-237A, Cambridge MA 02139, USA

## Abstract

**Background:**

Microarrays are widely used to investigate the blood stage of *Plasmodium falciparum *infection. Starting with synchronized cells, gene expression levels are continually measured over the 48-hour intra-erythrocytic cycle (IDC). However, the cell population gradually loses synchrony during the experiment. As a result, the microarray measurements are blurred. In this paper, we propose a generalized deconvolution approach to reconstruct the intrinsic expression pattern, and apply it to *P. falciparum *IDC microarray data.

**Methods:**

We develop a statistical model for the decay of synchrony among cells, and reconstruct the expression pattern through statistical inference. The proposed method can handle microarray measurements with noise and missing data. The original gene expression patterns become more apparent in the reconstructed profiles, making it easier to analyze and interpret the data. We hypothesize that reconstructed gene expression patterns represent better temporally resolved expression profiles that can be probabilistically modeled to match changes in expression level to IDC transitions. In particular, we identify transcriptionally regulated protein kinases putatively involved in regulating the *P. falciparum *IDC.

**Results:**

By analyzing publicly available microarray data sets for the *P. falciparum *IDC, protein kinases are ranked in terms of their likelihood to be involved in regulating transitions between the ring, trophozoite and schizont developmental stages of the *P. falciparum *IDC. In our theoretical framework, a few protein kinases have high probability rankings, and could potentially be involved in regulating these developmental transitions.

**Conclusions:**

This study proposes a new methodology for extracting intrinsic expression patterns from microarray data. By applying this method to *P. falciparum *microarray data, several protein kinases are predicted to play a significant role in the *P. falciparum *IDC. Earlier experiments have indeed confirmed that several of these kinases are involved in this process. Overall, these results indicate that further functional analysis of these additional putative protein kinases may reveal new insights into how the *P. falciparum *IDC is regulated.

## Introduction

Approximately 40% of the global population is at risk for contracting malaria, and an estimated 780,000 people die annually from this disease [1]. Human malaria is caused by five *Plasmodium *species, of which *P. falciparum *is responsible for the majority of human fatalities. The disease is transmitted when an infected mosquito bites a person, and injects sporozoites that migrate to and develop in the liver before merozoites are released into the bloodstream and invade red blood cells (RBCs) [2]. Within the RBC, *P. falciparum *undergoes a well-defined developmental cycle (IDC) during a 48-hour period that is characterized by three main stages, namely: rings, trophozoites and schizonts [2]. Schizont-infected RBCs rupture at the end of this cycle to release merozoites that can invade RBCs and reestablish a new IDC. In addition to the morphologic changes that characterize parasite development during the IDC, changes in gene expression accompany [3,4] and most likely drive this developmental program.

Gene expression during the 48-hour IDC has been densely profiled at 1-hour intervals using microarray technology in an effort to understand how overall gene expression patterns help shape blood stage parasite biology [3,4]. These studies revealed that the levels of many transcripts are reproducibly high or low at characteristic times within the IDC. Genes involved in key biological processes most relevant to a given IDC stage are generally coordinately up- and down- regulated. In fact, a 'just-in-time' model to describe transcriptionally regulated gene expression in *P. falciparum *has been proposed [4,5]. Here, a transcript's level is proposed to peak just prior to when its encoded protein product is most critically needed. Regulation of general biological processes such as metabolism, DNA synthesis, protein turnover and red blood cell invasion, for example, is well-described by this model [3,4]. While the full complement of parasite proteins controlling IDC progression has not been identified, the just-in-time principle could be useful for identifying key, transcriptionally regulated proteins that play important roles in regulating this process.

Protein kinases represent one such protein class, and have previously been implicated in regulating various aspects of the cell cycle and development in *P. falciparum *[6-8]. The genome encodes a predicted 85 [9] or 99 [10] protein kinases, which include an expanded and divergent FIKK family, of which there are 20 members [9,11]. In *Plasmodium spp*., several protein kinases have been shown to be important in regulating blood stage biology, including parasite egress from infected RBCs [6-8]. Recently, a large-scale knockout screening effort identified 36 out of the 65 protein kinases evaluated (no FIKKs included) as likely essential to the development of *P. falciparum *blood stage parasites [12]. Overall, these studies highlight the important contribution of the protein kinases to regulating critical aspects of *P. falciparum *biology during the IDC.

Therefore, we have been interested in addressing whether computationally applying the just-in-time principle to publicly available microarray data for *P. falciparum *is a reasonably efficient strategy for identifying protein kinases that are important to transition through the IDC. Such an approach, if successful, could prioritize protein candidates for more exhaustive experimental analysis. Two fundamental assumptions underlie our analytical framework, namely: (1) an important subset of protein kinases regulating transition through the IDC is transcriptionally regulated, and this is captured in the publicly available microarray data; and (2) increases in protein kinase transcript levels predictably precede peak protein synthesis, the latter defining the time at which a given protein kinase plays its critical role in IDC progression. Our approach requires explicitly addressing the confounding factor of decaying synchrony of parasite cultures in order to improve the reliability of predictions. For microarray experiments, parasite cultures are initially synchronized. However, these cultures gradually lose synchrony over the experimental course [3]. Consequently, gene expression levels at discrete time points for individual parasites are not directly inferred from the observed microarray data, which reflect an ensemble of increasingly asynchronous parasite transcription profiles. To address this issue, we introduce a deconvolution approach for reconstructing the intrinsic gene expression pattern of individual parasites from microarray data. This work was partly presented in our previous paper [8]. We identify and account for three main factors driving asynchrony in gene expression profiles during a microarray experiment, namely: (1) diversity of infection time; (2) diversity of growth rate; and (3) the emergence of early stage parasites intermixed with later stage parasites. By including these considerations into our computational framework, we are able to more accurately reconstruct single cell, global gene expression profiles, from which the temporal expression profile for individual protein kinases are determined.

## Methods

A publicly available microarray data set on three *P. falciparum *strains (HB3, 3D7 and Dd2) is used in this work [4,13]. In the HB3 data set, the expression profiles of 4345 oligonucleotide sequences have been measured at 48 time points with 1 hour interval. The 23^rd ^and 29^th ^data points are missing for all oligonucleotide sequences. The oligonucleotide sequences associated with protein kinases involved in the *P. falciparum *life cycle are retrieved from the PlasmoDB database. Some protein kinases have several unique oligonucleotide sequences. For those protein kinases, an average trace is calculated from the curves associated with each oligonucleotide sequence. In this fashion, the gene expression profiles of 65 protein kinases are collected from the data set of HB3. Along the same lines, 52 protein kinases and 51 protein kinases are collected from the data set 3D7 and Dd2 respectively. In the rest of this section, we present a computational method to extract the intrinsic gene expression pattern from the microarray data.

Gene expression levels obtained in microarray experiments are aggregates across many individual iRBCs. First, we will derive a set of linear equations (15) that relates the microarray data to the intrinsic expression pattern of individual iRBC. Next, by solving the corresponding linear inverse problem (16), we reconstruct the expression pattern. All symbols used in this paper are explained in the Table 1.

**Table 1:** Explanations of symbols

Symbols	Explanations
*M*	the total amount of cells in the media, it consists of RBCs and iRBCs
*S*(*t*)	the number of schizonts which infect RBCs at time *t*
*R*(*t*)	the number of fresh RBCs infected by schizont at time *t*
*a*_in_	the average number of RBCs infected by one schizont during the infection period
*a*_af_	the average number of RBCs infected by one schizont after the infection period
$\tilde{L}$	the normalized life span of individual iRBCs
$p_{\tilde{L}}\left(l\right)$	the probability density function of normalized life span $\tilde{L}$
*L*	the average life span of iRBCs
*L*'	the individual life span of iRBCs
ℓ	the cell age of iRBC in hours
{*f_i_*(ℓ), ℓ ∈ [0, *L*]}	the intrinsic gene expression pattern of protein *i *
ℓ_re_	the rescaled cell age according to its normalized life span $\tilde{L}$
{*f_i_*(ℓ_re_), $\ell_{\text{re}}=\tilde{L}\ell$, ℓ ∈ [0, *L*']}	the gene expression profile of individual iRBC on protein *i*
*S_f _*(*t*)	the number of fast-growing iRBCs which infect RBCs at time *t*
*R_f _*(*t*)	the number of RBCs infected by fast-growing iRBCs at time *t *
*N*(*t*)	the total number of iRBCs that have been infected at time *t *
N(*t*, ℓ_re_)	the number of iRBCs which reach rescaled cell age ℓ_re _at time *t *
*e_i_*(*t*)	the observed expression level of protein *i *at time *t *
*${\tilde{f}}_{i}\left(\ell \right)$*	the normalized gene expression pattern on protein *i*
*s*	the transitions between three stages of iRBC: ring, trophozoite, and schizont
*L_i_(s)*	the likelihood that protein kinases *i *is involved in regulating the stage transition *s *
*T*_s_	the time point when stage transition *s *occur
*n *	the average number of merozoites released by one schizont
*p *	the probability that one merozoites does not infect any RBC
*V *	the volume of merozoites can travel after it is released from schizont
*V*_total_	the volume of whole media
*m *	the total number of RBCs in the media

### Analysis of decaying synchrony

We assume that three main factors drive the iRBCs out of synchronization in the microarray experiment, namely: (A) diversity of infection time, (B) diversity of growth rate, and (C) the emergence of early stage parasites intermixed with later stage parasites. These are discussed below.

#### Diversity of infection time

**Figure 1:**
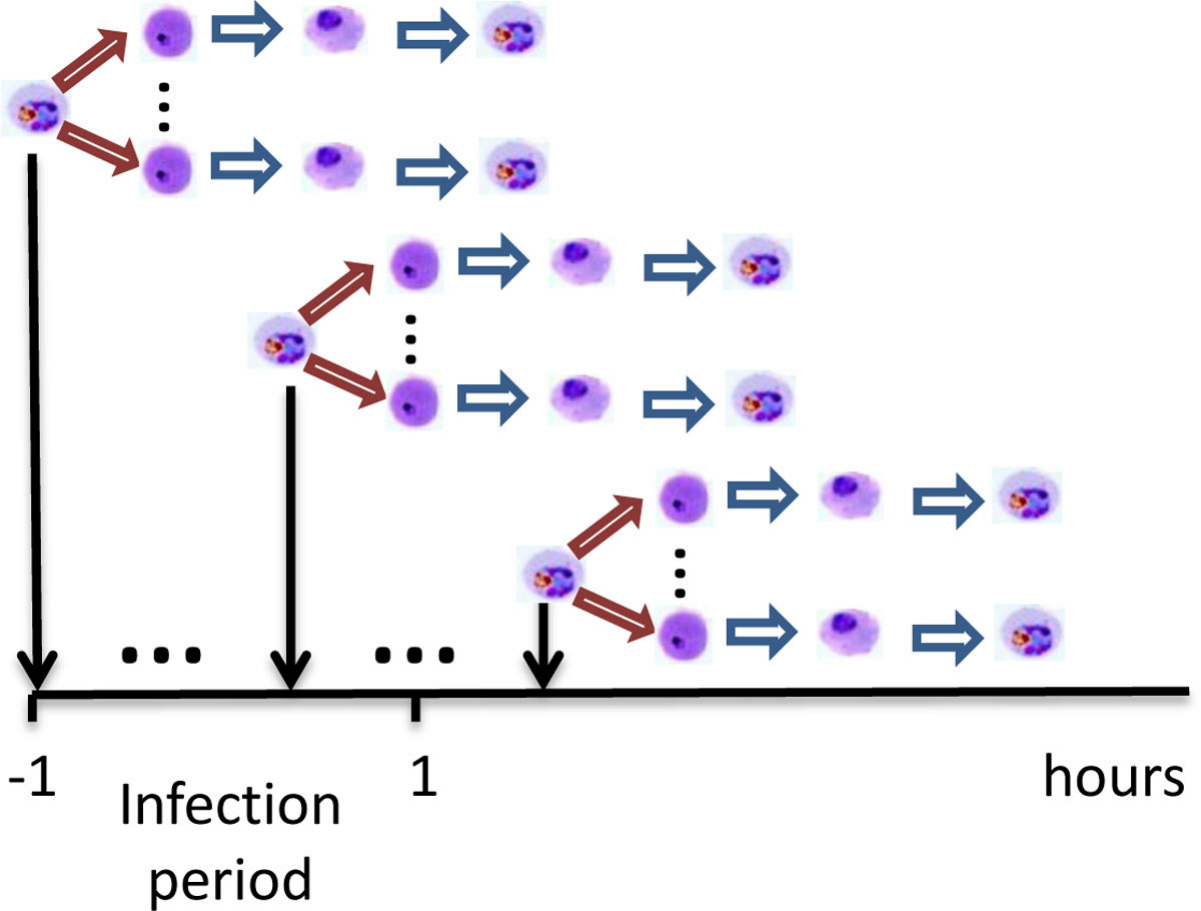
**Diversity of infection time among iRBCs**. The invasion of RBCs does not occur simultaneously. RBCs will be continuously infected as long as schizonts remain.

As illustrated in Figure 1, the invasion of RBCs does not occur simultaneously. In the microarray experiment [4], late-stage schizonts are synchronized by six sorbitol treatments on three generations. Prior to the first microarray time point, fresh RBCs are infected by late-stage schizonts within two hours, raising the parasitemia from 5% to 16%. After the invasion period, 80% of parasites are in the ring stage. Let *M *stand for the total number of cells in the media. In other words, 5% × *M *of schizonts infect 80% × 16% × *M *of ring during two-hour invasion, remaining 20% × 16% × *M *of schizonts are still alive after the invasion period. Although the concentration of RBCs is reduced from 14% to 3.3% immediately after the invasion period, RBCs can still be infected as long as schizonts remain. Therefore, a large amount of RBCs will be infected after the two-hour invasion.

Let *R*(*t*) denote the number of fresh RBCs infected by schizont at time *t *(hours). In the perfectly synchronized case, *R*(*t*) should be a Dirac delta function, which means all iRBCs are simultaneously infected at the same time. In microarray experiment, however, *R*(*t*) has a high value during the invasion period, and it maintains positive as long as schizonts remain.

Let *S*(*t*) stand for the number of schizonts which infect RBCs at time *t*. The parameters *a*_in _and *a*_af _denote the average number of RBCs infected by one schizont during and after the infection period respectively. Therefore, the expression of *R*(*t*) can be written as:

(1) $$R\left(t\right)=\left\{\begin{array}{c} a_{\text{in}}S\left(t\right),\;\;\text{if}\;\text{t}\;\in \;\;\text{[infection period],}\\ a_{\text{af}}S\left(t\right),\;\;\text{if}\;\text{t}\;\in \;\;\text{[after infection period]}\cdot \end{array}\right)$$

At the end of this section, we explain how to estimate the parameters *a*_in_, *a*_af _and function *S*(*t*).

#### Diversity of growth rate

**Figure 2:**
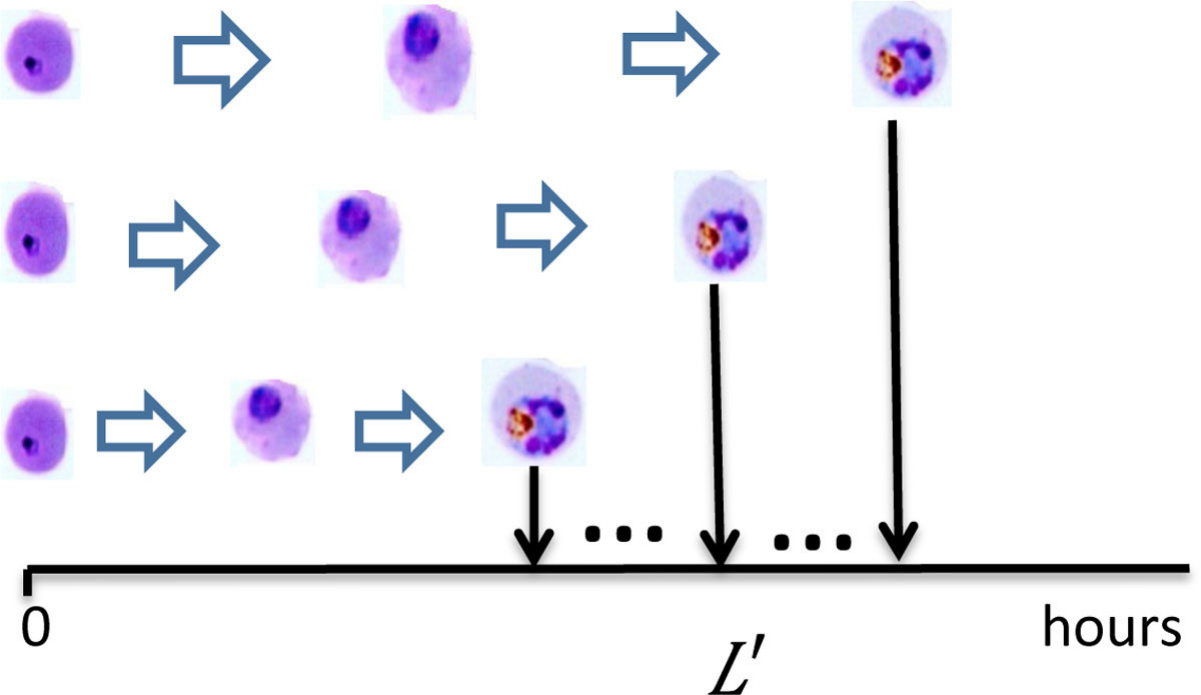
**Diversity of growth rate among iRBCs**. The iRBCs grow at different rates.

The iRBCs grow at different rates [4]. Consequently, as illustrated in Figure 2, synchrony gradually decays, even if all iRBCs are simultaneously infected at the same time. Let the random variable $\tilde{L}$ indicate the normalized life span of iRBC, which is the quotient of individual life span *L*' and the average life span *L*:

(2) $$\tilde{L}=\frac{L^{\prime }}{L}.$$

$\tilde{L}$ is assumed to follow a normal distribution: $\tilde{L}\sim N\left(1,\sigma^{2}\right).$ Hence the probability density function $p_{\tilde{L}}\left(l\right)$of normalized life span $\tilde{L}$ can be written as:

(3) $$p_{\tilde{L}}\left(l\right)=\frac{1}{\sqrt{2\pi \sigma^{2}}}e^{-\frac{{\left(l-1\right)}^{2}}{2\sigma^{2}}},$$

where the value of *σ *is adjusted to fit the experimental observations. The details will be discussed later in this paper.

Let {*f_i_*(ℓ), ℓ ∈ [0, *L*]} be the intrinsic gene expression pattern of protein *i *on one complete life span, where ℓ denotes the cell age of iRBC (hours). Since *f_i_*(ℓ) represents the common pattern shared by individual RBC, the expression profile of individual iRBC is assumed to be $\left\{f_{i}\left(\ell_{\text{re}}\right),\ell_{\text{re}}=\ell /\tilde{L},\ell \in \left[0,L^{\prime }\right]\right\}$, where ℓ_re _denotes the rescaled cell age according to its normalized life span $\tilde{L}$ For instance, one iRBC has been infected for ℓ hours, its current expression level on the protein *i *is written as $f_{i}\left(\ell /\tilde{L}\right),$ where $\tilde{L}$ is the normalized life span of the corresponding RBC.

#### The emergence of early stage parasites intermixed with later stage parasites

**Figure 3:**
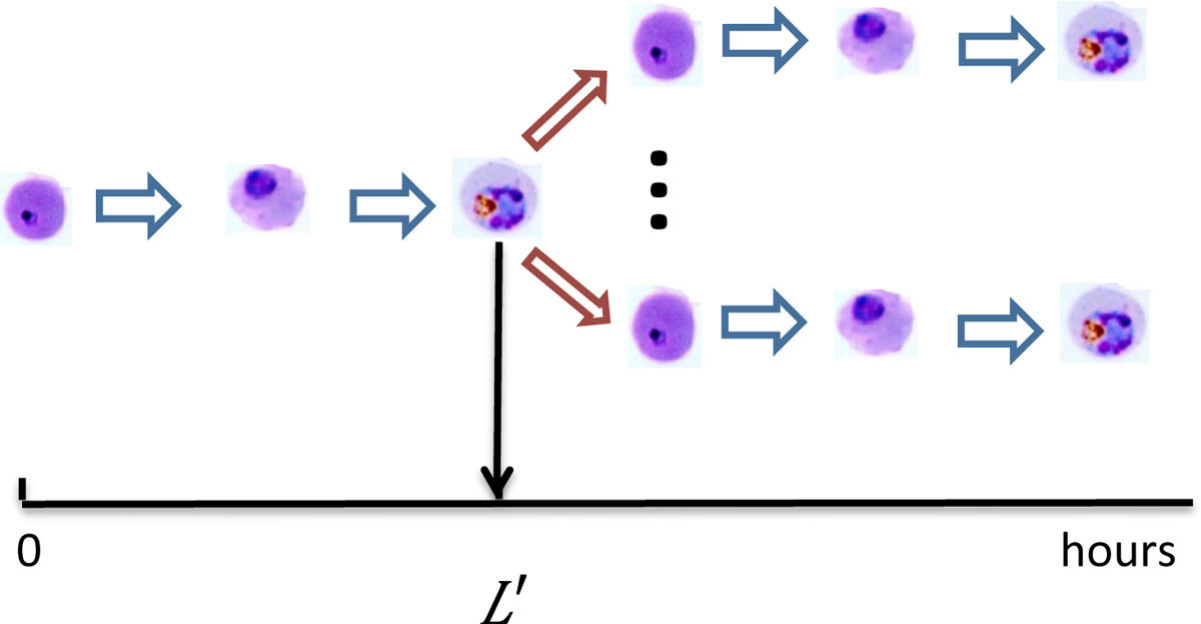
**The emergence of early stage parasites intermixed with later stage parasites**. Additional fresh RBCs will be infected by fast-growing iRBCs which reach the late stage of schizont early.

Due to the diversity of growth rate, a few iRBCs can reach the late stage of schizont early. As a result, those fast-growing iRBCs can infect additional fresh RBCs, as illustrated in Figure 3. This phenomenon has been observed in experiments [4]. Let *S_f _*(*t*) denote the number of fast-growing iRBCs which reach end of their life span at time *t*. Let *R_f _*(*t*) be the number of RBCs which are infected by these iRBCs at time *t*. We assume that *R_f _*(*t*) is proportional to *S_f _*(*t*):

(4) $$R_{f}\left(t\right)=a_{\text{af}}S_{f}\left(t\right).$$

The invasion factor *a*_af _stands for the average number of fresh RBCs that will be infected by one schizont after the invasion period.

As shown in (2), the normalized life span $\tilde{L}$ stands for the quotient of individual life span *L*' and the average life span *L*. The number of iRBCs that start and end their life span at time *t *are denoted as *R*(*t*) and *S_f_*(*t*) respectively. Given the probability density function $p_{\tilde{L}}\left(l\right)$ of normalized life span $\tilde{L}$, the number of iRBCs that have reached the end of their life span at time *t *can be written as follow:

(5) $$\begin{array}{llll} \int_{-\infty }^{t}S_{f}\left(t^{\prime }\right)\text{d}t^{\prime } & =\int_{-\infty }^{+\infty }R\left(t^{\prime }\right)P_{\tilde{L}}\left(\tilde{L}<\frac{t-t^{\prime }}{L}\right)\text{d}t^{\prime }\; & & \;\\ & =\int_{-\infty }^{+\infty }R\left(t^{\prime }\right)\int_{-\infty }^{\frac{t-t^{\prime }}{L}}p_{\tilde{L}}\left(l\right)\text{d}l\text{d}t^{\prime }.\; & & \;\\ & \; & \end{array}$$

Therefore, the expression of *S_f _*(*t*) can be derived from (5) as:

(6) $$\begin{array}{llll} S_{f}\left(t\right) & =\frac{\text{d}}{\text{d}t}\int_{-\infty }^{t}S_{f}\left(t^{\prime }\right)\text{d}t^{\prime }\; & & \;\\ & =\frac{1}{L}\int_{-\infty }^{+\infty }R\left(t^{\prime }\right)p_{\tilde{L}}\left(\frac{t-t^{\prime }}{L}\right)\text{d}t^{\prime }.\; & & \;\\ & \; & \end{array}$$

Therefore, we have the expression of *R_f _*(*t*) by substituting (6) into (4):

(7) $$R_{f}\left(t\right)=\frac{a_{\text{af}}}{L}\int_{+\infty }^{+\infty }R\left(t\prime \right)p_{\tilde{L}}\left(\frac{t-t\prime }{L}\right)\text{d}t\prime .$$

### Simulation of iRBCs population distribution

**Figure 4:**
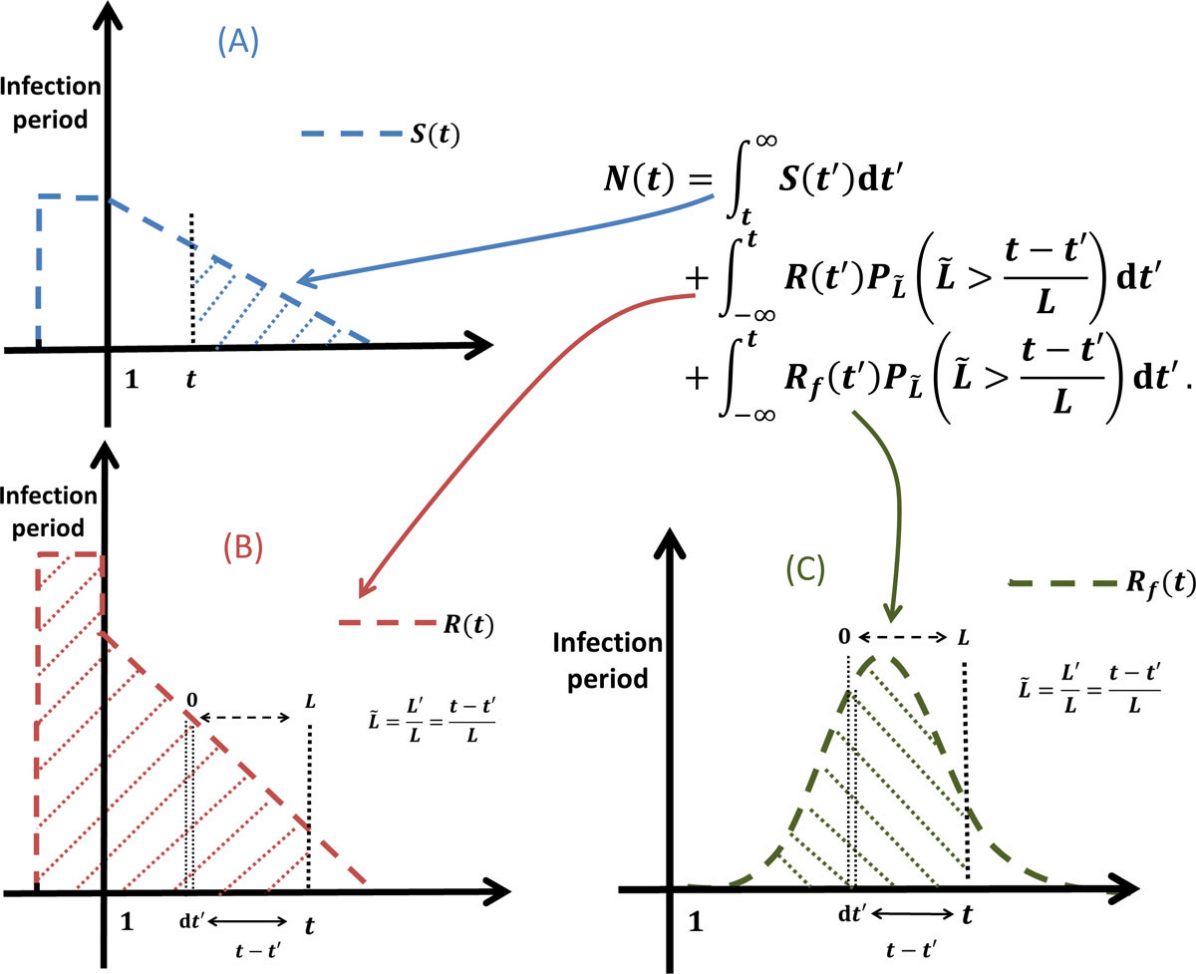
**The total number of iRBCs ***N*(*t*) **at time ***t*****. (A) The late-schizonts that infect fresh RBCs during infection period and have not yet burst at time *t*. (B) The first generation of iRBCs (infected by late-schizonts around infection period) that have not yet reached the end of their life span at time *t*. (C) Second generation of iRBCs (infected by fast-growing iRBCs) that have not yet reached the end of their life span at time *t*.

Let *N*(*t*) denote the total number of iRBCs at time *t*. *N*(*t*) consists of 3 parts: the late-schizonts that infect fresh RBCs during the infection period and have not yet burst at time *t*, the first generation of iRBCs (infected by late-schizonts around infection period) that have not yet reached the end of their life span at time *t*, and second generation of iRBCs (infected by fast-growing iRBCs) that have not yet reached the end of their life span at time *t*. Therefore, as illustrated in Figure 4, we can decompose *N*(*t*) as:

(8) $$N\left(t\right)=\int_{t}^{+\infty }S\left(t^{\prime }\right)\text{d}t^{\prime }+\int_{+\infty }^{t}R\left(t^{\prime }\right)p_{\tilde{L}}\left(\tilde{L}>\frac{t-t^{\prime }}{L}\right)\text{d}t^{\prime }+\int_{+\infty }^{t}R_{f}\left(t^{\prime }\right)p_{\tilde{L}}\left(\tilde{L}>\frac{t-t^{\prime }}{L}\right)\text{d}t^{\prime },$$

where *S*(*t*) stands for the number of late-schizonts bursts at time *t*, *R*(*t*) denotes the number of RBCs infected by late-schizonts at time *t*, and *R_f _*(*t*) is the number of RBCs which are infected by fast-growing iRBCs at time *t*. The expressions of *R*(*t*) and *R_f _*(*t*) are given by (1) and (7) respectively. The expression of *S*(*t*) will be derived in (25).

**Figure 5:**
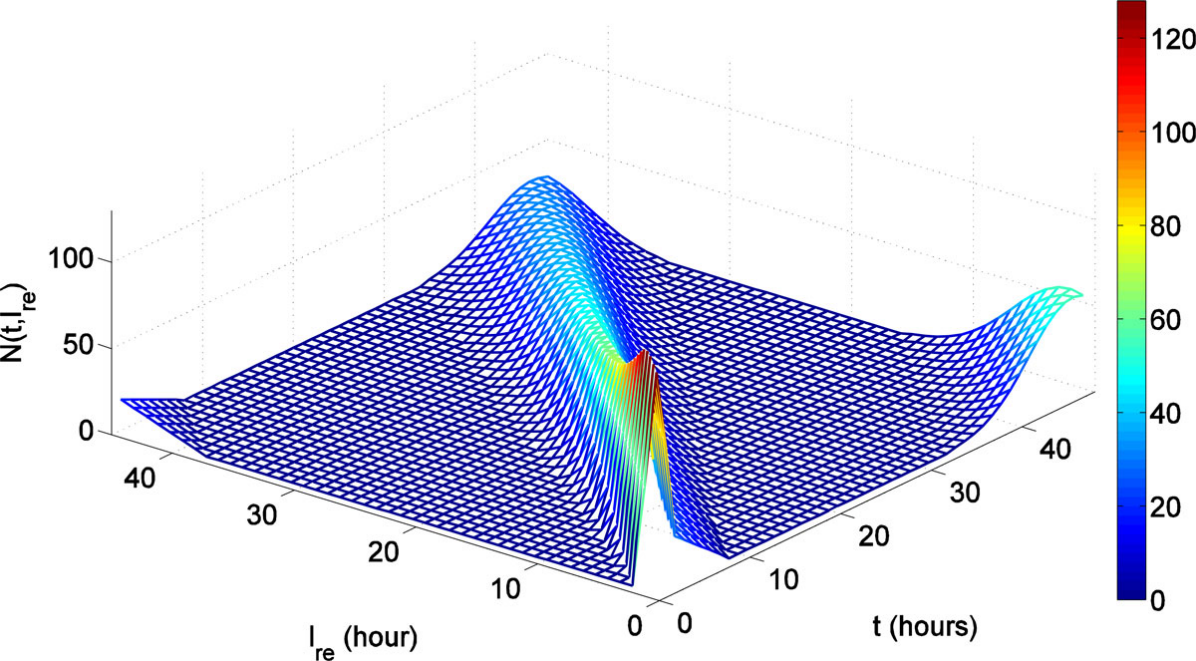
**iRBCs population distributions change over time**. N(*t*, ℓ_re_) with *σ *= 0.1. It indicates the number of iRBCs that reach the rescaled cell age ℓ_re _at time *t*.

Let *N*(*t*, ℓ_re_) stand for the number of iRBCs that reach the rescaled cell age ℓ_re _at time *t*. In other words, given the time t, *N*(*t*, ℓ_re_) indicates the distribution of iRBCs over a complete life span of iRBC (see Figure 5). Therefore, *N*(*t*, ℓ_re_) and *N*(*t*) satisfy following relation:

(9) $$N\left(t\right)=\int_{-\infty }^{L}N\left(t,{\ell^{\prime }}_{\text{re}}\right)\text{d}{\ell^{\prime }}_{\text{re}}.$$

**Figure 6:**
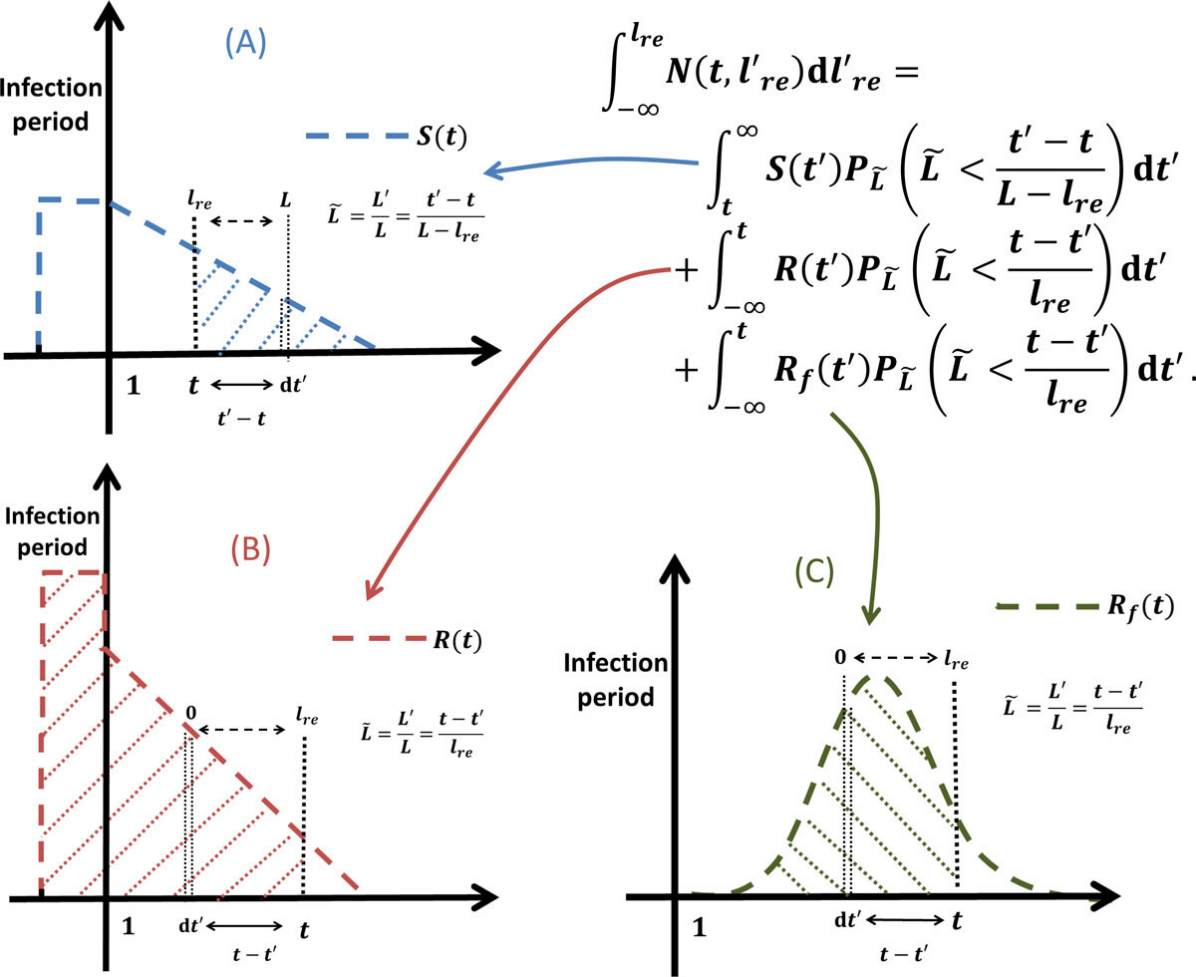
**The number of iRBCs that have not reached the rescaled cell age ℓ_re _at time **t****. (A) The late-schizonts that infect fresh RBCs around infection period and have not reached the rescaled cell age ℓ_re _at time t. (B) The first generation of iRBCs that have not reached the rescaled cell age ℓ_re _at time t. (C) Second generation of iRBCs that have not reached the rescaled cell age ℓ_re _at time *t*.

Hence, as illustrated in Figure 6, the number of iRBCs that have not yet reached the rescaled cell age ℓ_re _time *t *can be expanded from (8) as follows:

(10) $$\begin{array}{ccc} \int_{-\infty }^{\ell_{\text{re}}}N\left(t,{\ell^{\prime }}_{\text{re}}\right)\text{d}{\ell^{\prime }}_{\text{re}} & = & \int_{t}^{+\infty }S\left(t^{\prime }\right)P_{\tilde{L}}\left(\tilde{L}<\frac{t^{\prime }-t}{L-\ell_{\text{re}}}\right)\text{d}t^{\prime }+\int_{-\infty }^{t}R\left(t^{\prime }\right)P_{\tilde{L}}\left(\tilde{L}>\frac{t-t^{\prime }}{\ell_{\text{re}}}\right)\text{d}t^{\prime }\\ & + & \int_{-\infty }^{t}R_{f}\left(t^{\prime }\right)P_{\tilde{L}}\left(\tilde{L}>\frac{t-t^{\prime }}{\ell_{\text{re}}}\right)\text{d}t^{\prime }\\ & = & \int_{t}^{+\infty }S\left(t^{\prime }\right)\int_{-\infty }^{\frac{t^{\prime }-t}{L-\ell_{\text{re}}}}p_{\tilde{L}}\left(\ell \right)\text{d}l\text{d}t^{\prime }+\int_{-\infty }^{t}R\left(t^{\prime }\right)\int_{\frac{t-t^{\prime }}{\ell_{\text{re}}}}^{{}^{+\infty }}p_{\tilde{L}}\left(l\right)\text{d}l\text{d}t^{\prime }\\ & + & \int_{-\infty }^{t}R_{f}\left(t^{\prime }\right)\int_{\frac{t-t^{\prime }}{\ell_{\text{re}}}}^{+\infty }p_{\tilde{L}}\left(l\right)\text{d}l\text{d}t^{\prime }. \end{array}$$

Therefore, *N*(*t*, ℓ_re_) can be derived from (10) as follows:

(11) $$\begin{array}{llll} N\left(t,\ell_{\text{re}}\right) & =\frac{\text{d}}{\text{d}\ell_{\text{re}}}\int_{-\infty }^{\ell_{\text{re}}}N\left(t,{\ell^{\prime }}_{\text{re}}\right)\text{d}\ell_{\text{re}}^{{}^{\prime }}\; & & \;\\ & =\int_{t}^{+\infty }S\left(t^{\prime }\right)p_{\tilde{L}}\left(\frac{t^{\prime }-t}{L-\ell_{\text{re}}}\right)\frac{t^{\prime }-t}{{\left(L-\ell_{\text{re}}\right)}^{2}}\text{d}t^{\prime }+\int_{-\infty }^{t}R\left(t^{\prime }\right)p_{\tilde{L}}\left(\frac{t-t^{\prime }}{\ell_{\text{re}}}\right)\frac{t-t^{\prime }}{{\ell_{\text{re}}}^{2}}\text{d}t^{\prime }\; & & \;\\ & +\int_{-\infty }^{t}R_{f}\left(t^{\prime }\right)p_{\tilde{L}}\left(\frac{t-t^{\prime }}{\ell_{\text{re}}}\right)\frac{t-t^{\prime }}{{\ell_{\text{re}}}^{2}}\text{d}t^{\prime }.\; & & \;\\ & \; & \end{array}$$

By substituting the expression of *R_f _*(*t*) (7) into (11), *N*(*t*, ℓ_re_) can be written as:

(12) $$\begin{array}{llll} N\left(t,\ell_{\text{re}}\right) & =\int_{t}^{+\infty }S\left(t^{\prime }\right)p_{\tilde{L}}\left(\frac{t^{\prime }-t}{L-\ell_{\text{re}}}\right)\frac{t^{\prime }-t}{{\left(L-\ell_{\text{re}}\right)}^{2}}\text{d}t^{\prime }+\int_{-\infty }^{t}R\left(t^{\prime }\right)p_{\tilde{L}}\left(\frac{t-t^{\prime }}{\ell_{\text{re}}}\right)\frac{t-t^{\prime }}{{\ell_{\text{re}}}^{2}}\text{d}t^{\prime }\; & & \;\\ & +\frac{a_{\text{af}}}{L}\int_{-\infty }^{t}p_{\tilde{L}}\left(\frac{t-t^{\prime }}{\ell_{\text{re}}}\right)\frac{t-t^{\prime }}{{\ell_{\text{re}}}^{2}}\text{d}t^{\prime }\int_{-\infty }^{+\infty }R\left(t^{\prime \prime }\right)p_{\tilde{L}}\left(\frac{t^{\prime }-t^{\prime \prime }}{L}\right)\text{d}t^{\prime \prime }.\; & & \;\\ & \; & \end{array}$$

### Modeling of gene expression level

The gene expression levels obtained in microarray experiments are aggregates across many individual iRBCs. This superposition across iRBCs is modeled by means of a linear system. Let *e_i_*(*t*) denote the observed expression level of protein *i *at time *t*. As discussed earlier, *N*(*t*, ℓ_re_) denotes the distribution of iRBCs on rescaled cell age ℓ_re _and $\left\{f_{i}\left(\ell_{\text{re}}\right),\ell_{\text{re}}=\ell /\tilde{L},\ell \in \left[0.L^{\prime }\right]\right\}$ stands for the gene expression level of individual iRBC according to its rescaled cell age ℓ_re_. Therefore, the observed expression level *e_i_*(*t*) can be written as a integral over one complete life span of iRBCs as follows:

(13) $$e_{i}\left(t\right)=\int_{0}^{L}N\left(t,\ell_{\text{re}}\right)f_{i}\left(\ell_{\text{re}}\right)\text{d}\ell_{\text{re}}.$$

We represent the continuous function *f_i_*(*s*) and *N*(t, ℓ_re_) as a series of discrete points {*f_i_*(1), *f_i_*(2),..., *f_i_*(*T*)}, and {*N*(*t*,1), *N*(*t*,2),...,*N*(*t*,T)} respectively. Therefore, *e_i_*(*t*) can be approximated as:

(14) $$e_{i}\left(t\right)\approx \overset{L}{\underset{\ell_{\text{re}}=1}{\sum }}N\left(t,\ell_{\text{re}}\right)f_{i}\left(\ell_{\text{re}}\right)\Delta \ell_{\text{re}}.$$

In microarray experiments, gene expression levels are measured at a series of discrete time points. Hence, the resulting observed expression level *e_i_*(*t*) is a series of discrete value. In the dataset HB3, for example, the expression levels are measured every hour over a period of 48 hours. Therefore, a linear system can be derived based on (14):

(15) $$\underset{A}{\underset{︸}{\left(\begin{array}{c} N\left(1,1\right)\;N\left(1,2\right)\;\;\ldots \;N\left(1,L\right)\\ N\left(2,1\right)\;N\left(2,2\right)\;\;\ldots \;N\left(2,L\right)\\ \vdots \;\vdots \;\ddots \;\vdots \; \end{array}\right)}}\;\underset{x}{\underset{︸}{\left(\begin{array}{c} f_{i}\left(1\right)\\ f_{i}\left(2\right)\\ \;\vdots \\ f_{i}\left(L\right) \end{array}\right)}}=\underset{b}{\underset{︸}{\left(\begin{array}{c} e_{i}\left(1\right)\\ e_{i}\left(2\right)\\ \;\vdots \end{array}\right)}}.$$

The observation matrix *A *can be calculated by means of the equation (12). The element of matrix *A *at row *t *and column ℓ_re _denotes the number of iRBCs that reach the rescaled cell age *l*_re _at time point *t*. The constant vector *b *stands for the gene expression levels observed in microarray experiment. The unknown variable vector *x *is the intrinsic gene expression pattern of individual cell. We can find *x *by solving the discrete linear inverse problem (15).

### Reconstruction of synchronized expression profile

For each protein *i*, the observed expression level *e_i _*is modeled as the superposition of the expression level *f_i _*of individual iRBCs, as described in (15). To solve the described linear inverse problem (15), we minimize an objective function. The objective function contains the squared error (*Ax *- *b*)(*Ax *- *b*) ^⊤^. The intrinsic gene expression pattern of iRBCs is assumed to be a smooth curve. Therefore, we also include square difference as $\sum_{k=1}^{L-1}{\left(x_{k}-x_{k+1}\right)}^{2}+{\left(x_{L}-x_{1}\right)}^{2}$ in the objection function. We also need to impose the constraint *x *≥ 0 because expression levels are positive. In summary, we compute the intrinsic gene expression pattern *x *as follows:

(16) $$x=\underset{x\geq 0}{\text{arg}\text{min}}\left\{\underset{\text{square error}}{\underset{︸}{\left(Ax-b\right)\;{\left(Ax-b\right)}^{⊤}}}+c\underset{\text{gradients}}{\underset{︸}{\left[\overset{L-1}{\underset{k=1}{\sum }}{\left(x_{k}-x_{k+1}\right)}^{2}+{\left(x_{L}-x_{1}\right)}^{2}\right]}}\right\}.$$

We solve (16) numerically by means of the function *f mincon *in the Optimization Toolbox of Matlab (MATLAB 7.9, The MathWorks Inc.)

### Prediction of protein kinases

As we discussed earlier, an increased gene expression level before a cell stage transition is regarded as a sign that the corresponding protein kinase is involved in that stage transition. We denote the likelihood that protein kinases *i *is involved in regulating the stage transition *s *by *L_i_*(*s*). Let *T_s _*stand for the time point when stage transition *s *occur. The expression of likelihood *L_i_*(*s*) is derived in following.

Let ${\tilde{f}}_{i}\left(\ell \right)$ be the normalized expression pattern such that its integral over one iRBC life span is equal to 1:

(17) $${\tilde{f}}_{i}(\ell )=\frac{f_{i}(\ell )}{\sum_{\ell =1}^{L}f_{i}(\ell )\Delta l},$$

where ℓ = 1,..., *L*. Since protein kinases are often regulated at translational and post-translational levels [5], *L_i_*(*s*) is estimated as the maximum sum of *W *consecutive points of ${\tilde{f}}_{i}\left(\ell \right)$ that appear *H *hours prior to *T_s_*.

(18) $$L_{i}\left(s\right)=\underset{T_{s}-H\leq \ell_{1}\leq T_{s}-W+1}{\text{max}}\left\{\overset{\ell_{1}+W-1}{\underset{\ell =\ell_{1}}{\sum }}{\tilde{f}}_{i}\left(\ell \right)\right\}.$$

Consequently, protein kinase *i *can be prioritized by its likelihood *L_i_*(*s*) of being involved in a stage transition.

### Parameter estimation

The proposed method for reconstructing expression patterns consists of two main steps. First, the linear system described in (15) is built by calculating the cell age distribution *N*(*t*, ℓ_re_) (12) for each element of the observation matrix *A*. Second, the expression pattern is reconstructed by solving the discrete linear inverse problem (16). Therefore, the performance of our method mainly depends on the calculation of *N*(*t*, ℓ_re_). As shown in equation (12), *N*(*t*, ℓ_re_) is dominated by three sets of parameters: the infection factors *a*_in _and *a*_af_, the burst rate of schizonts *S*(*t*), and the standard deviation *σ *of normalized life span $\tilde{L}$.

**Figure 7:**
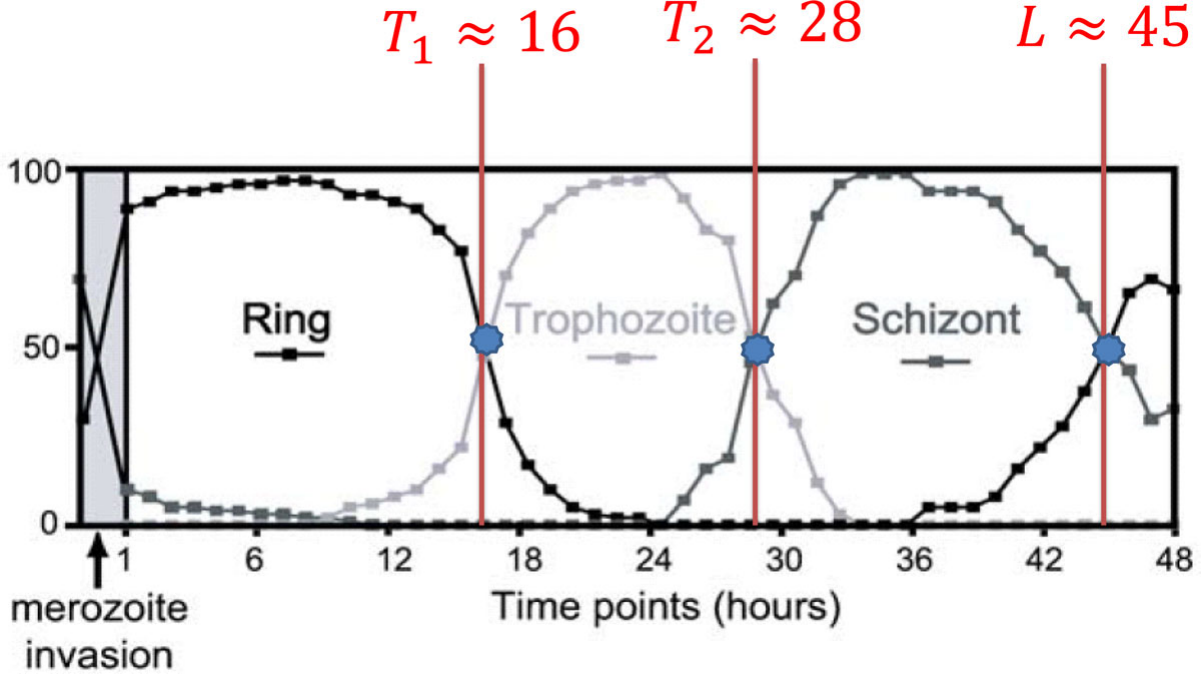
**Percent representation of iRBCs observed in experiment**. The boundaries (*T*_1_, *T*_2 _and *L*) between the three stages (ring, trophozoite, and schizont) are approximated from the percent representation of iRBCs observed at every time point in experiment [4].

The infection factors *a*_in_, *a*_af_, and burst rate of schizonts *S*(*t*) can be accurately estimated from parasitemia and percent representation of iRBC observed at each time point. As shown in Figure 7, the percent representation of iRBC is available at each time point [4]. However, the parasitemia is given only at two time points, one during and one after the infection period. In this section, we explain how we estimate all parameters from the specification of the microarray experiments [4].

#### Infection factors

In the proposed model, the infection factors, *a*_in _and *a*_af _denote the number of RBC that can be infected by one bursted schizont during the infection period and after the period respectively. As discussed earlier, 5% × *M *schizonts infect 80% ×16% × *M *of ring cell during invasion period, and the remaining 20% × 16% × *M *schizonts are still alive after the invasion period. Therefore, the value of *a*_in _can be deduced as follows:

(19) $$a_{\text{in}}=\frac{16\%\times 80\%\times M}{5\%\times M-16\%\times 20\%\times M}\approx 7.11.$$

After the invasion period, the cell concentration (both fresh and infected) is reduced from 14% to 3.3%. To estimate the value of *a*_af_, we propose a simple model to describe how infection factor is influenced by the cell concentration.

At the end of the life span, the cell membrane of schizont bursts, and merozoites are released to infect other RBCs [14]. We denote by *p *the probability that a merozoite does not infect any RBC, and let *n *represent the average number of merozoites released by one schizont. The value of *n *is estimated to be 14, because one schizont usually contains 12 to 16 merozoites [14]. Therefore, the average number of RBCs infected by one bursted schizont is given by *n*(1 - *p*).

In the following, we derive an expression for the probability *p*. First, we assume that each merozoite can travel a certain space after it is released from schizont, and let *V *be the volume of this space. Second, we also assume that if a RBC appears in the space indicated by *V*, it will be immediately infected by the corresponding merozoite. Let *V*_total _denote the volume of whole media. Let *m *stand for the total number of RBCs remaining in the media. Hence, the value of *p *can be estimated as the probability that none of the *m *RBCs appear in the space *V*. If the RBCs are uniformly distributed in *V*_total_, it follows:

(20) $$p={\left(\frac{V_{\text{total}}-V}{V_{\text{total}}}\right)}^{m}.$$

The concentration of RBCs is reduced by adjusting the volume of culture *V*_total _from 1000 milliliter to 4500 milliliter after the infection period [4]. Therefore, the expression of *a*_in _and *a*_af _can be obtained by substituting (20) into *n*(1 - *p*) as follows:

(21) $$\left\{\begin{array}{c} a_{\text{in}}=14\left(1-{\left(\frac{1000-V}{1000}\right)}^{m}\right)\\ a_{\text{af}}=14\left(1-{\left(\frac{4500-V}{4500}\right)}^{m^{\prime }}\right). \end{array}\right)$$

At the beginning of the experiment, the culture is initialized by 115.0 milliliter of purified RBC [4]. Human blood has 4 to 6 million RBC per microliter (cubic millimeter), and the corresponding hematocrit is about 45% [15]. Hence, the number of RBC *m *before the infection period can be estimated as:

(22) $$m=\frac{5,000,000\times 115,000}{45\%}\approx 1.28\times 10^{12}.$$

After the infection period, the parasitemia is raised from 5% to 16%. Therefore, the number of RBC before the infection period *m *and after the infection period *m*' satisfy following relation:

(23) $$\frac{m}{100\%-5\%}=\frac{m^{\prime }}{100\%-16\%}.$$

Consequently, the number of RBC after the infection period *m' *can be estimated. By substituting the value of *a*_in_, *m *and *m*' into (21), the value of *V *and *a*_af _can be deduced as follows:

(24) $$\left\{\begin{array}{c} V\approx 5.55\times 10^{-10}\text{millilitre,}\\ a_{\text{af}}\approx 1.82. \end{array}\right)$$

The derivation of *V *is related to the concept of mean free paths in physics, roughly the diffusion length for the first binding event or the lifetime [16].

#### Burst rate of schizonts

**Figure 8:**
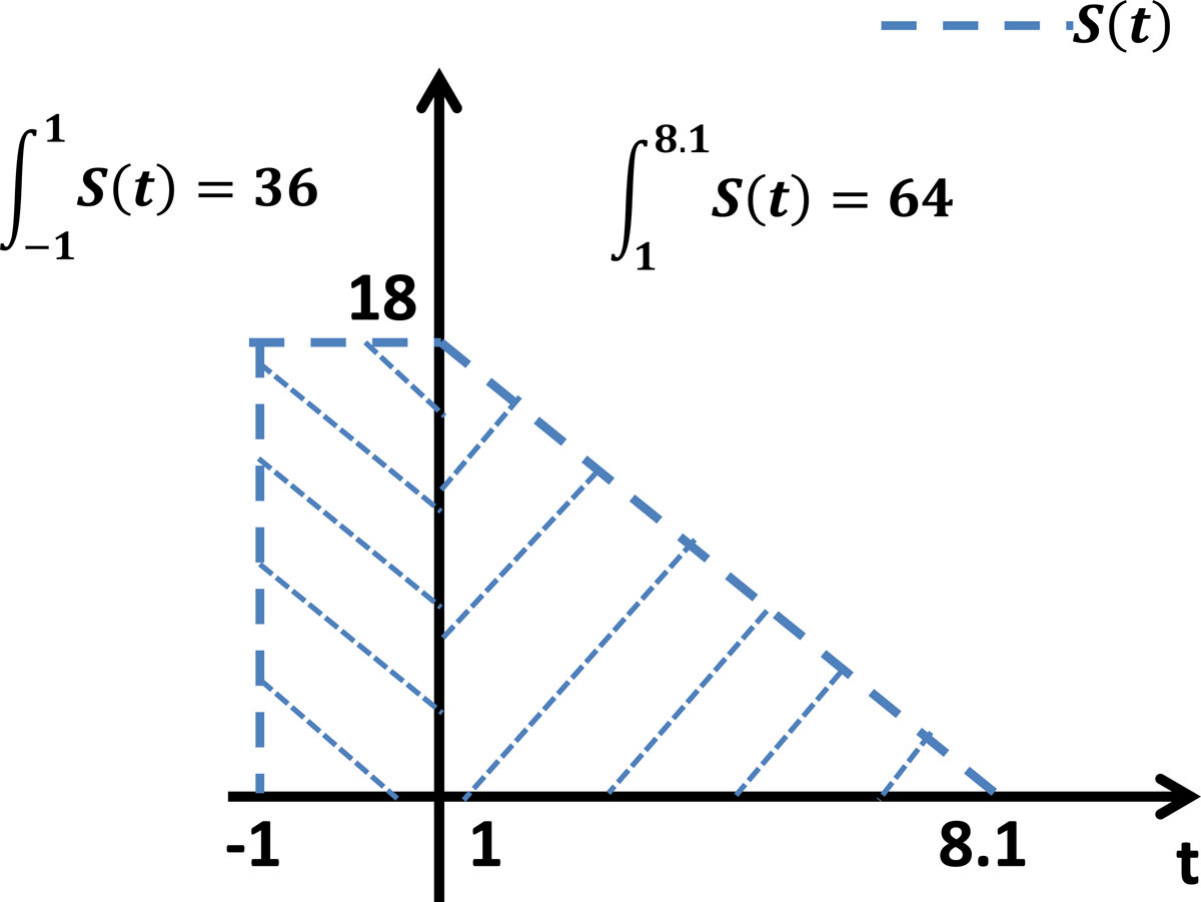
**Burst rate of schizonts**. The number of schizonts *S*(*t*) infecting RBCs at time *t *is approximated as a piecewise linear function according to experimental observations.

The number of schizonts infecting RBCs at time *t *is denoted by *S*(*t*). As discussed earlier, 5% × *M *- 16% × 20% × *M *schizonts burst in the two-hour infection period, leaving 16% × 20% × *M *schizonts alive till around 7 hours after the invasion period (8th microarray data point). In other words, 36% of schizonts burst in the first two hours followed by the remaining 64% of schizonts which burst within the next 7 hours. Therefore, *S*(*t*) is approximated as a piecewise linear function whose integral on these two periods is 36 and 64 respectively. The value of *S*(*t*) in the first two hours is also assumed to be a constant. As shown in Figure 8, consequently, the expression of *S*(*t*) can be written as follows:

(25) $$s\left(t\right)=\left\{\begin{array}{c} 18,\\ -2.54t+20.54,\\ 0, \end{array}\begin{array}{c} \text{if}\;-1\leq t<1,\\ \text{if}\;1\leq t\leq 8.1,\\ \text{otherwise}\text{.} \end{array}\right)$$

#### Standard deviation of normalized life span

Given *a*_in_, *a*_af_, and *S*(*t*), the iRBCs population distributions *N*(*t*, ℓ_re_) (12) depend on the probability density function $p_{\tilde{L}}$(*l*) of normalized life span $\tilde{L}$ (3). The normalized life span $\tilde{L}$ has been assumed to follow a normal distribution. By definition, the mean of $\tilde{L}$ is equal to 1. The standard deviation *σ *is estimated such that the resulting iRBCs population distributions *N*(*t*, ℓ_re_) are in agreement with the experimental data.

Over one complete life cycle, iRBCs go through three life stages, i.e., ring, trophozoite and schizont, as follows:

(26) $$\text{iRBC}\;\text{stage}=\left\{\begin{array}{c} \text{ring,}\\ \text{trophozoite,}\\ \text{schizont,} \end{array}\begin{array}{c} \text{if}\;\text{0}\leq \ell_{\text{re}}<T_{1},\\ \text{if}\;T_{1}\leq \ell_{\text{re}}<T_{2},\\ \text{if}\;T_{2}\leq \ell_{\text{re}}\leq L. \end{array}\right)$$

As illustrated in Figure 7, the value of *T*_1_, *T*_2 _and *L *are approximated from the percent representation of iRBCs. Given the boundaries between the three stages, iRBCs population distributions *N*(*t*, ℓ_re_) can represent the percent representation of iRBCs at each time point as follows. By definition, *N*(*t*, ℓ_re_) stands for the number of iRBCs that reach the rescaled cell age *l_re _*at time *t*. Hence, the number of iRBC at a specific stage (ring, trophozoite, and schizont) can be estimated as a integral of *N*(*t*, ℓ_re_) on the rescaled cell age *l_re_*. Therefore, the percent of ring cell at time *t *can be calculated as follows:

(27) $$\text{percent of ring at time }t\text{ = }\frac{\overset{\text{ring}}{\overset{︷}{\int_{0}^{T_{1}}N\left(t,\ell_{\text{re}}\right)\text{d}\ell_{\text{re}}}}}{\underset{\text{ring}}{\underset{︸}{\int_{0}^{T_{1}}N\left(t,\ell_{\text{re}}\right)\text{d}\ell_{\text{re}}+}}\underset{\text{trophozoite}}{\underset{︸}{\int_{T_{1}}^{T_{2}}N\left(t,\ell_{\text{re}}\right)\text{d}\ell_{\text{re}}+}}\underset{\text{schizont}}{\underset{︸}{\int_{T_{2}}^{L}N\left(t,\ell_{\text{re}}\right)\text{d}\ell_{\text{re}}.}}}$$

The percent of trophozoite and schizont can be calculated similarly.

**Figure 9:**
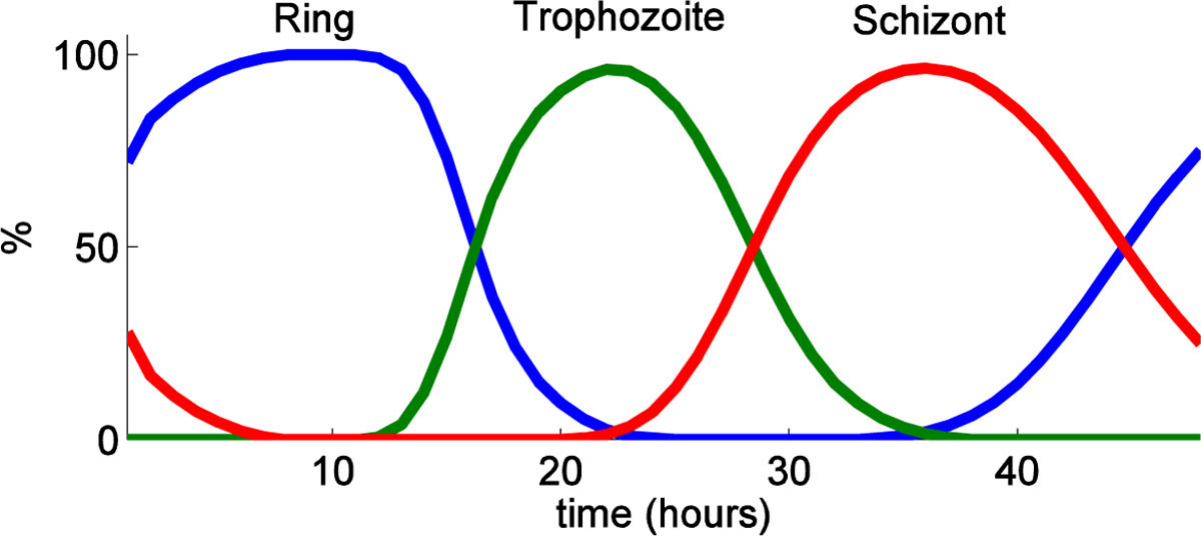
**Percent representation of iRBCs calculated from iRBCs population distributions**. The percent of iRBCs (ring trophozoite, and schizont) calculated from iRBCs population *N*(*t*, ℓ_re_) with *σ *= 0.1.

The distribution *N*(*t*, ℓ_re_) for *σ *= 0.1 is shown in Figure 5. The percent of ring, trophozoite, and schizont calculated from *N*(*t*, ℓ_re_) is illustrated in Figure 9. The corresponding calculated percent representation of iRBCs depends on the value of *σ*. Consequently, we tune the value of *σ *such that percent representation observed in experiment, as illustrated in Figure 7, coincides with the simulated percent representation, as illustrated in Figure 9.

## Results on synthetic data

In this section, the proposed method is evaluated on synthetic microarray data.

### Generate synthetic microarray data

**Figure 10:**
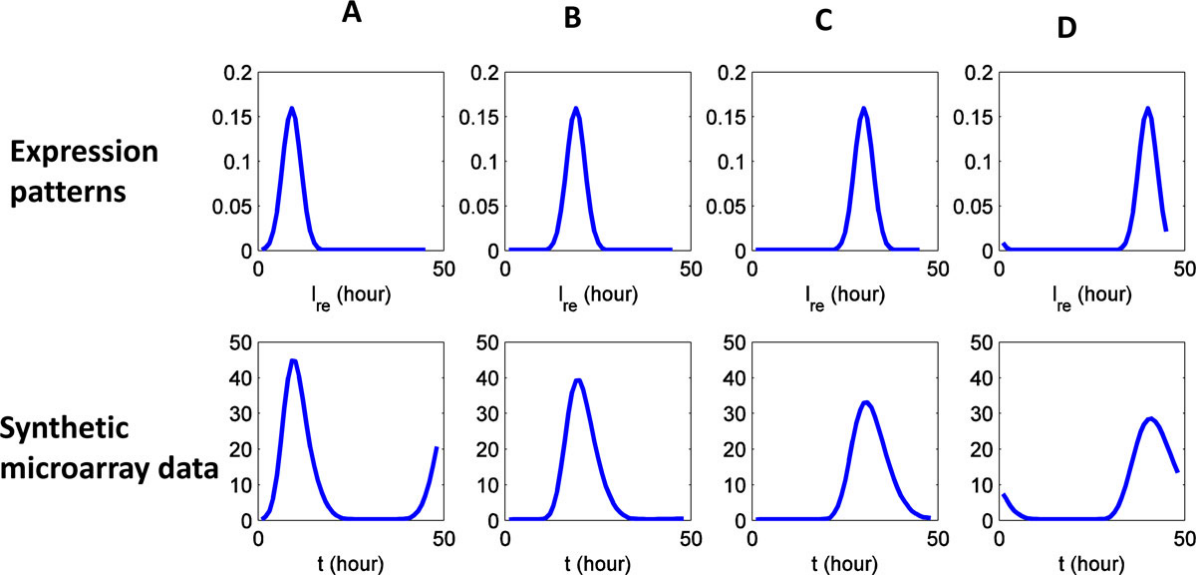
**Synthetic microarray data generated from known expression patterns**. Synthetic microarray data are generated for four expression patterns (A, B, C and D).

The data obtained in microarray experiments are aggregates across many cells. This superposition across cells is modeled by means of a linear system (15). The matrix *A *in that linear system depends on the parameters (*a*_in_, *a*_af_, *σ*, and *S*(*t*)), estimated from microarray experiments [4]. Synthetic microarray data is generated by substituting the expression pattern of individual iRBC *x *into the linear system. As shown in Figure 10, we generate synthetic microarray data for four expression patterns (A, B, C and D). Each of them simulates a protein kinase that serves a specific function in cell stage transition, and hence has high expression level in a short time period before a cell stage transition.

The microarray experiments measure the superposition across cells. Due to the decay of synchrony among cells, the intrinsic expression pattern is blurred in microarray data. By comparing the intrinsic expression patterns and synthetic microarray data shown in Figure 10, we demonstrate how decaying synchrony can blur the expression pattern.

### Tolerance to signal noise and missing data points

Due to the assay complexity in microarray experiments, signal noise [17] and missing time points [18] are common phenomena observed in real microarray data. To evaluate the performance of our method under similar conditions, expression patterns are reconstructed from synthetic microarray data contaminated by Gaussian white noise and/or missing data points. The resulting expression patterns are compared to the original expression patterns (A, B, C and D).

**Figure 11:**
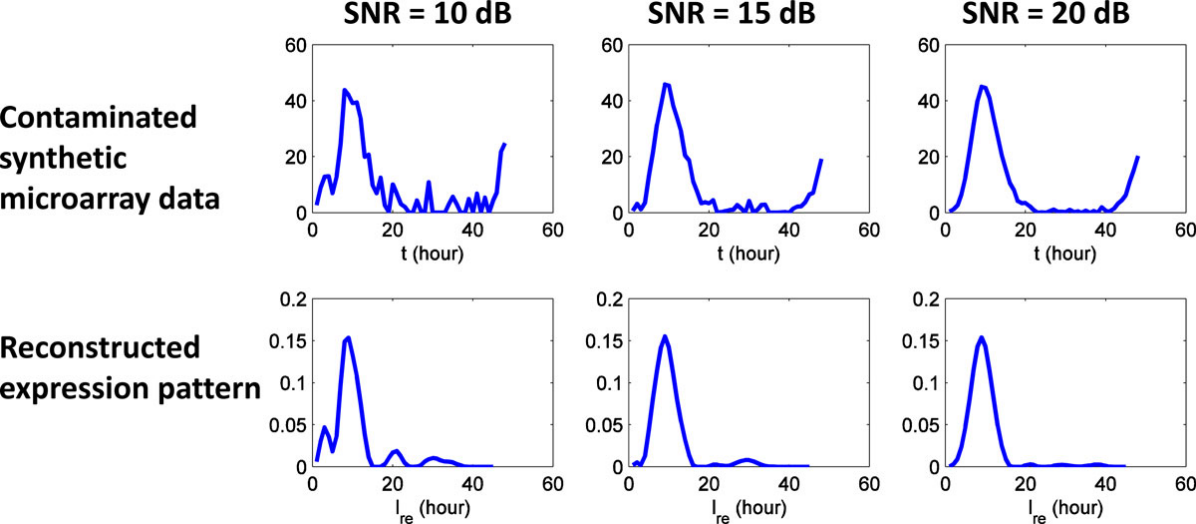
**Microarray data contaminated by Gaussian white noise**. Gaussian white noise is used to contaminate the synthetic microarray data of expression pattern (A). The reconstructed patterns are quite similar to the original expression pattern (A).

As shown in Figure 11, the synthetic microarray data of expression pattern (A) are contaminated by Gaussian white noise. The signal to noise ratios (SNR) of the resulting microarray data are 10, 15 and 20 dB respectively. To calculate matrix *A*, we choose the same parameter values (*a*_in_, *a*_af_, *σ*, and *S*(*t*)) as used to generate synthetic microarray data. In the next section, we will investigate how the results vary with other choice of those parameter values. The resulting matrix *A *is substituted into the linear system described in equation (15), and the expression pattern is reconstructed by solving the corresponding linear inverse problem (16). The resulting expression patterns shown in Figure 11 are quite similar to the original expression pattern (A) given in Figure 10.

**Figure 12:**
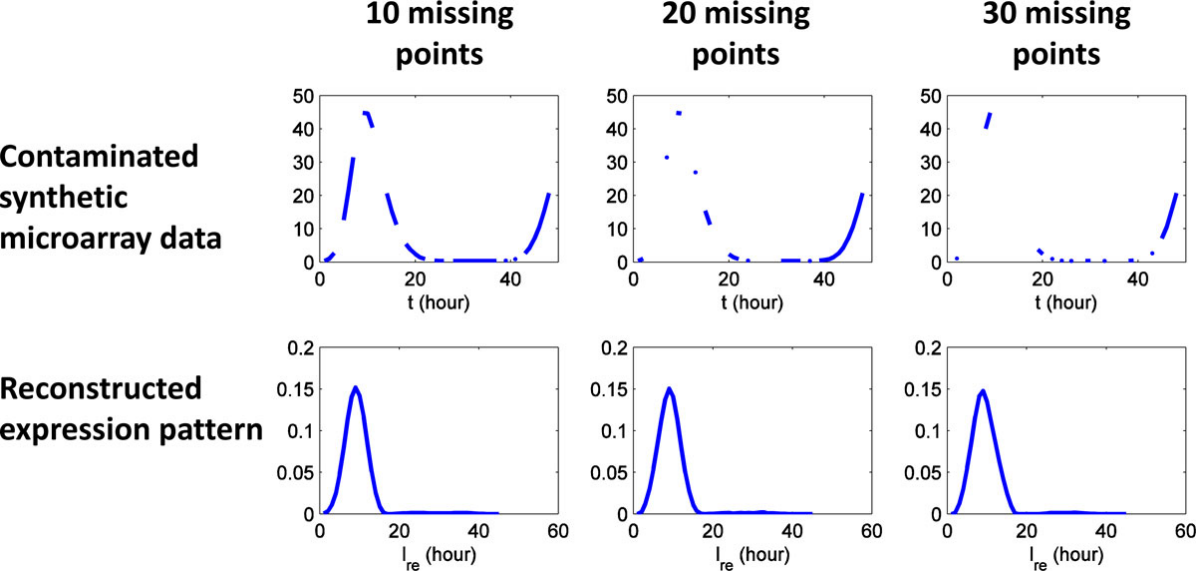
**Microarray data contaminated by random missing data points**. Data points (10, 20 and 30) are randomly remove from the microarray data of expression pattern. The expression pattern can be reliably reconstructed, even when 30 of 48 time points are missing in the microarray data.

Along the same lines, the tolerance to missing data points is evaluated using synthetic microarray data with missing data points. We randomly remove data points (10, 20 and 30) from the microarray data of expression pattern respectively. Then expression pattern are reconstructed from contaminated microarray data. As shown in Figure 12, the proposed method generates a reliable estimate for the original expression pattern (A), even when 30 of 48 time points are missing in the microarray data.

**Figure 13:**
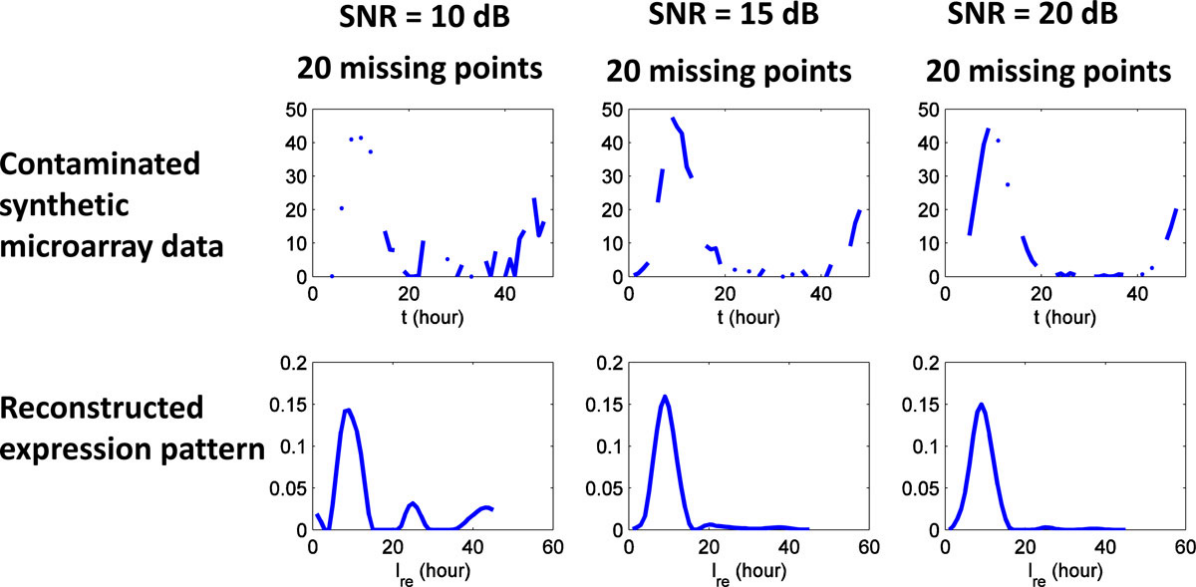
**Microarray data contaminated by both Gaussian white noise and random missing data points**. The proposed method can reliably reconstruct the expression pattern when the microarray data is simultaneously contaminated by both signal noise (SNR = 10, 20, and 30 dB) and missing data points (20).

Real microarray data are usually contaminated by both signal noise and missing data points. Therefore, as shown in Figure 13, we conduct additional experiments on synthetic microarray data contaminated by both Gaussian white noise (SNR = 10, 20, and 30 dB) and 20 missing data points. The results of Figure 13 suggest that the proposed method can reliably reconstruct the expression pattern when the microarray data is simultaneously contaminated by both signal noise and missing data points.

### Sensitivity to model parameters

The performance of our method mainly depends on the calculation of cell age distribution *N*(*t*, ℓ_re_). As shown in equation (12), *N*(*t*, ℓ_re_) depends on three sets of parameters: the infection factors *a*_in _and *a*_af_, the bufirst rate of schizonts *S*(*t*), and the standard deviation *σ *of normalized life span $\tilde{L}$. In the experiments of the previous section, the same parameters are used to generate synthetic data and to reconstruct expression patterns. However, in real microarray data, these parameters are unknown, and need to be estimated from experimental observations. The infection factors *a*_in_, *a*_af_, and burst rate *S*(*t*) of schizonts can be accurately calculated from parasitemia and percent representation of iRBC. It is difficult to precisely estimate the standard deviation *σ *of normalized life span $\tilde{L}$. In this section, we investigate how results vary with regards to the choice of *σ*.

**Figure 14:**
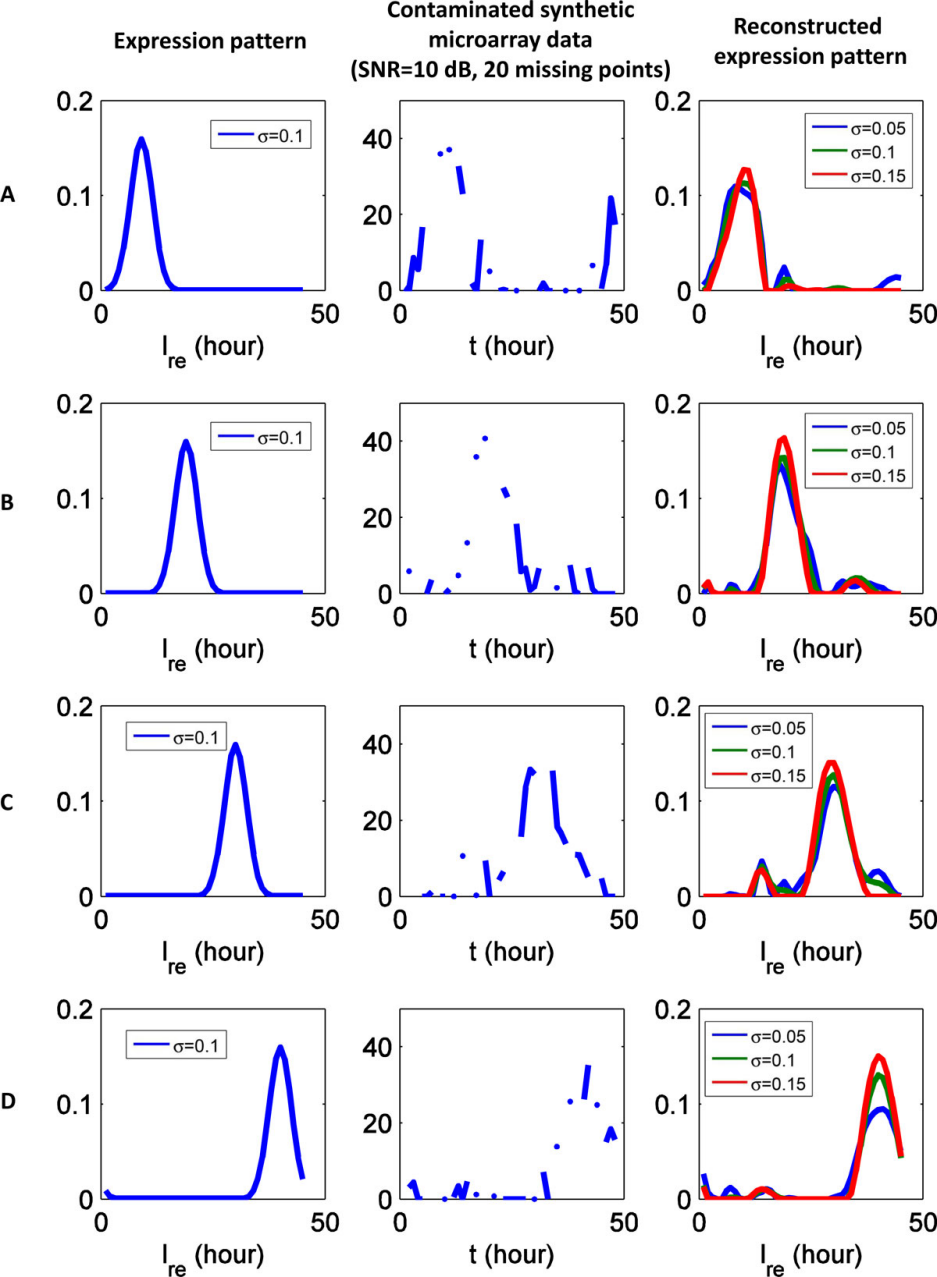
**Sensitivity to model parameter ***σ*****. Synthetic microarray data is generated from know expression pattern with *σ *= 0.1. The synthetic microarray data is contaminated by Gaussian white noise (SNR = 10 dB) and 20 missing data points. The reconstructed expression patterns only slightly change as the value of *σ *varies (*σ *=0.05, 0.1, or 0.15).

Synthetic microarray data is generated from know expression pattern with *σ *= 0.1. The synthetic microarray data is contaminated by Gaussian white noise (SNR = 10 dB) and 20 missing data points. The expression pattern is reconstructed with various values of *σ *(*σ*=0.05, 0.1, or 0.15). As shown in Figure 14, the reconstructed expression patterns only slightly change as the value of *σ *varies. Consequently, the reconstruction is robust to the choice of *σ*.

## Results on real data

Expression patterns are reconstructed for 68 protein kinases collected from the microarray data of *P. falciparum *(HB3, 3D7 and Dd2) [4,13]. Reconstructed expression patterns are substituted into the equation (18) to estimate the likelihood of each protein kinase being associated with a specific IDC transition, and hence, contributing to effecting either the stage transition itself or a process(es) needed in the subsequent stage. Data for the three broad transitions analyzed, namely ring-to-trophozoite, trophozoite-to-schizont and schizont-to-ring, are summarized in Additional Files 1, 2 and 3, respectively.

A primary motivation for developing this computational framework is to prioritize gene candidates with potentially important stage-dependent functions for detailed downstream experimental analysis of gene function. In the ring-to-trophozoite analysis, several members of the largely unstudied FIKK protein kinases emerge with relatively high probabilities of mediating important biology during this developmental transition. Several of these protein kinases are targeted to the infected RBC cytosol/membrane [19]. Two [MAL7P1.144, PFL0040c] have been previously studied using gene knockout approaches [20]. While non-essential to blood stage parasite growth and survival, these proteins help mediate the increased rigidity of infected RBCs observed in trophozoite stage parasites. Presumably, this requires modulation of the RBC cytoskeleton through a combination of RBC cytoskeletal and/or exported parasite protein phosphorylation and increased interactions between these [20]. The analysis here suggests that the other highly ranked family members (see Additional File 1) could also be mediating important yet unknown biology at this ring-trophozoite transition.

Interestingly, in the trophozoite-schizont and schizont-ring analyses, a number of protein kinases previously established to be essential and in some cases implicated in directly impacting the transition emerge with the highest probability rankings (Additional Files 2 and 3) [6-8,12]. In the schizont-ring analysis, for example, PFB0815 has previously been implicated in parasite motility/invasion/egress [6], and PF13_-_0211 in parasite egress from the RBC [7]. Furthermore, MAL13P1.278 is an essential Aurora kinase (Pfark1) that associates with spindle pole bodies during parasite schizogeny and is implicated in cell cycle regulation [8]. Overall, the top ranked genes in the trophozoite-schizont and schizont-ring analyses are highly enriched for protein kinases previously established to be essential to parasite survival. Therefore, it will be intriguing to experimentally examine the other highly ranked genes in both transition categories [PFI1415w, PF11_-_0079, PF10_-_0380, PF11_-_0510, PFI0095c, PFE0045c and PFC0945w] as these may also play critical roles in regulating parasite development during these stages and could provide new opportunities for antimalarial drug development.

## Conclusions

This study proposes a new methodology to reconstruct intrinsic expression patterns from microarray data. We derive a linear system that relates the microarray data to the expression patterns. By solving the corresponding linear inverse problem, the expression patterns are reconstructed. The experiments conducted on synthetic data suggest that the proposed method can reliably reconstruct the expression pattern, even though both signal noise and missing data points contaminate the microarray data. By applying this method to *P. falciparum *microarray data, protein kinases are prioritized in terms of their likelihood of being involved in regulating some aspect(s) of the IDC. Indeed, the results of our analyses are supported by previously published experimental data confirming the involvement of several protein kinases in regulating parasite biology at or between developmental stage transitions in the IDC. Our results indicate that experimentally investigating the function of other putative protein kinases not previously studied but ranked highly in our analysis may provide new insights into *P. falciparum *biology.

## Competing interests

The authors declare that they have no competing interests.

## Authors' contributions

Z.W. and J.D. prepared the first draft of the manuscript. J.N. proposed the initial idea, and helped with the data analysis and interpretation. Z.W., J.D., and J.C. conducted the theoretical analysis in this study. The numerical experiments were carried out by Z.W. All authors read and approved the final manuscript.

## Supplementary Material

Additional file 1 **Protein kinases are prioritized in terms of their likelihoods of being involved in the stage transition from ring to trophozoite**. Click here for file

Additional file 2 **Protein kinases are prioritized in terms of their likelihoods of being involved in the stage transition from trophozoite to schizont**. Click here for file

Additional file 3 **Protein kinases are prioritized in terms of their likelihoods of being involved in the stage transition from schizont to ring**. Click here for file
